# Acute effects of different resistance training loads on cardiac autonomic modulation in hypertensive postmenopausal women

**DOI:** 10.1186/s12967-018-1615-3

**Published:** 2018-08-30

**Authors:** Arthur F. Vale, Juliana A. Carneiro, Paulo C. V. Jardim, Thiago V. Jardim, James Steele, James P. Fisher, Paulo Gentil

**Affiliations:** 1 Programa de Pós Graduação em Ciência da Saúde, Universidade Federal de Goiás, Goiânia, Brazil; 2 Faculdade de Educação Física e Dança, Universidade Federal de Goiás, Campus Samambaia, Avenida Esperança S\N, Caixa Postal 131 Goiânia, Goiás Brazil; 3 Liga de Hipertensão Arterial, Universidade Federal de Goiás, Goiânia, Brazil; 4 Centre for Health, Exercise, and Sport Science, School of Sport, Health and Social Sciences, Southampton Solent University, Southampton, UK; 5 Ukactive Research Institute, London, UK

**Keywords:** Heart rate variability, Resistance training, Hypertension, Autonomic modulation

## Abstract

**Background:**

Individuals with arterial hypertension often have an autonomic nervous system (ANS) imbalance with predominance of sympathetic ANS. This predominance can lead to injury of several organs affecting its functioning. There is evidence that performing high intensity resistance training (RT) with heavier loads and a lower number of repetitions results in lower cardiovascular stress when compared with lighter loads and a higher number of repetitions. However, the effects of different protocols of RT in autonomic modulation are not known. Therefore, the aim of the study was to analyze and compare the effects of different protocols of high intensity of effort RT on autonomic cardiac modulation of hypertensive women.

**Methods:**

A randomized crossover design clinical trial was conducted with 15 postmenopausal hypertensive women who underwent a control session and two high intensity RT protocols involving 6 and 15 repetition maximum (RM). Heart rate variability (HRV), systolic blood pressure (SBP), diastolic blood pressure (DBP), heart rate (HR) and double product (DP) were collected pre, immediately post, 1 h post, and 24 h post each protocol. Repeated-measures ANOVA were used.

**Results:**

SBP was higher for 6RM than control immediately after session (p < 0.05). There were no differences for DBP among protocols (p ≥ 0.05). HR was higher for 15RM than 6RM and control immediately after and 1 h after session (*p *≤ 0.05). DP values for 15RM were significantly higher than 6RM and control immediately after the session and remained higher than control 1 h after session (*p *≤ 0.05). The indices that compose HRV (rMSSD) were lower after 15RM than 6RM and control (*p *≥ 0.05). The parameters of parasympathetic activity (HF) were decreased and sympathetic (LF) activity was increased for 15RM when compared to the 6RM and control session immediately after the exercise session (*p *≤ 0.05).

**Conclusion:**

Performing high intensity RT with lower loads and a higher number of repetitions seems to promote acute increases in sympathetic ANS activity, which may be related to cardiovascular stress. On the other hand, heavier load and lower repetition RT did not significantly impact upon autonomic modulation when compared to a control session.

## Background

Arterial hypertension (AH) is a multifactorial clinical condition characterized by elevated and sustained blood pressure (BP) levels reaching approximately 46% of the US population over 20 years [1]. AH is one of the most important public health problems and is considered one of the main risk factors for cardiovascular diseases [2, 3].

The autonomic nervous system (ANS) has an important role in regulating physiological processes both in normal and pathological conditions [4, 5]. Individuals with AH typically have an ANS imbalance with greater performance and predominance of sympathetic ANS [6–8]. In women, this sympathetic predominance is more pronounced with age, possibly as a result of the decrease in estrogen production, especially in the postmenopausal period, which favors the occurrence of AH [9, 10].

Among the techniques used to evaluate ANS activity, heart rate variability (HRV) is a simple and noninvasive measure of autonomic impulses, representing one of the most promising quantitative markers of autonomic modulation. In general, HRV describes the oscillations of the intervals between beats (R–R intervals) that are related to the influence of the ANS on the sinus node [4, 11, 12]. Recent studies showed that resistance training (RT) may promote positive adaptations in the ANS with a consequent increase in HRV as well as a chronic increase in muscle strength and a decrease in BP [13–16].

Whilst RT is considered effective in positively modulating the ANS, there is a shortage of studies evaluating the impact of different RT protocols on ANS. Understanding these responses would provide guidance for the most efficacious RT prescription for improvement in cardiovascular adaptation while decreasing other cardiovascular risk factors, particularly in populations such as those with AH who it may be advisable to avoid high levels of acute cardiovascular stress. Therefore, the aim of the study was to analyze and compare the effects of different protocols of high intensity of effort RT on hemodynamic parameters and autonomic cardiac modulation of postmenopausal hypertensive women.

## Methods

### Experimental approach to the problem

A randomized crossover study was conducted to compare cardiac autonomic modulation and other hemodynamic parameters in hypertensive women, aged 45–69 years. Tests were performed before, immediately after, 1 and 24 h after different protocols of resistance training with high intensity of effort. The study involved the comparison of three conditions: control, resistance training with lighter loads and higher number of repetitions (15 repetitions maximum, 15RM) and resistance training with heavier loads and a lower number of repetitions (six repetitions maximum, 6RM). The selection of participants was performed through the analysis of medical records at the University Hospital where preliminary data were obtained from the candidates for study participation. During the first contact via telephone, it was verified if the participants filled the participation criteria and the participants were invited to an initial visit for presentation, clarifications regarding the methodological procedures and any other doubts that could exist about the progress of the research. The study was approved by the Institutional Research Ethics Committee (Protocol 1,641,089).

### Participants

The sample consisted of 15 hypertensive and postmenopausal women, who were regularly enrolled in University Hospital care and who accepted to participate in the study. Exclusion criteria involved: current smoker or user of tobacco products; chronic alcoholism; body mass index (BMI) exceeding 35 kg/m^2^; hormone replacement therapy; beta-blocker use; use of anti-depressive and/or anxiolytic drugs; recent cardiovascular event such as acute myocardial infarction, stroke or heart failure (≤ 3 months); diabetes; heart failure and/or renal failure; musculoskeletal, untreated joint disease, or other incapacitating disease that could prevent the performance of the protocols. The drugs used to control AH were: diuretics (14 participants), angiotensin-converting enzyme inhibitors (7 participants) and angiotensin II receptor blockers (9 participants). Patients using any class of beta-blockers was excluded because it’s the only drug for AH control that interference on ANS responses in the different protocols [17, 18].

### Anthropometric measures

All anthropometric measures were performed using the World Health Organization standardization [19]. Body mass was measured using a portable electronic scale with a capacity of up to 200 kg and with a variation of 0.1 kg (OMRON HBF-214; OMRON Heath Care, Inc, Illinois, USA, 2013). The measurement was performed with the patient positioned in the center of the platform, without support and without making movements, in orthostatic posture, with arms hanging vertical alongside the body. Height was measured using a portable stadiometer with a variation of 0.1 cm (Seca Stadiometer; Seca GmBH & Co, Hamburg, Germany). The participants were instructed to stand barefoot, in an upright position, with their legs extended, feet parallel and heels together aligned with the door. BMI was calculated as the ratio between mass and the square of the participant’s height (kg/m^2^).

### Strength testing

Initial familiarization sessions were performed in order to adapt the participants to the practice and execution of the RT exercises. The exercises used were bench press, lat pull down and 45̊ leg press. The participants were individually supervised by at least two researchers on the correct execution of each exercise. During the familiarization session, each participant performed three sets of 12–15 repetitions with the minimum possible load in each exercise as warm-up. After 3-day intervals, the participants returned to University Hospital to perform the repetition maximum tests. The 15RM test started with a warm-up of 12 repetitions at a self-selected comfortable load. After warm-up, the participants performed up to five attempts with progressive load increases until the participant could not complete the 16th repetition for each of the proposed exercises. The participants were allowed to rest 5 min between each attempt. Following a period of at least 3 days each participant returned to the University Hospital for the 6RM test. The test started with a warm-up of 12 repetitions at a comfortable load. After the warm-up period, up to five attempts with progressive load increase were performed until the participant could not complete the 7th repetition for each of the proposed exercises. Five minutes of rest were allowed between each attempt.

### Experimental protocol

After determination of the loads of 6RM and 15RM, the participants performed three different experimental protocols: a control session, a RT session with 6RM, and a RT session with 15RM. The order of execution of the sessions was performed randomly by lot. A period of 3 days was given between each session. The protocols were performed at the same time of day (8–10 am) and in a room with controlled temperature (22 °C), in order to avoid the influence of the circadian cycle and external conditions. The participants were instructed to fast for 8 h, to avoid consumption of alcohol and stimulants (coffee, teas, soft drinks, etc.) 24 h before each test session, to not perform strenuous physical activities 48 h previous to the tests and to follow similar routines for all sessions. A food record was given to each participant to record the time, quantities and preparation of each food consumed the day before the first test. Then they were oriented to follow the same pattern in the days before the other sessions.

Upon arrival at the University Hospital the participants were referred to the clinical research laboratory and were advised to remain in the supine position for 10 min. After this period, systolic blood pressure (SBP) and diastolic blood pressure (DBP) were measured using an oscillometric device (OMRON, model HEM-705CP; OMRON Heath Care, Inc, Illinois, USA). Heart Rate (HR) and HRV were collected using a heart rate monitor (Polar^®^ V800, Electro Oi, Finland) using consecutive heart rate intervals (RR interval) for 10 min. During this period, the participants were oriented to remain in the supine position, avoid any movement, remain silent, do not sleep and maintain spontaneous breathing. After resting measures, participants were given 30 g of maltodextrin diluted in 300 mL of potable water.

The participants assigned to the sessions of 6RM or 15RM performed the three exercises already mentioned (lat pulldown, barbell bench press and 45° leg press) in three sets of 6RM or 15RM depending on the protocol chosen for the day. The exercises were selected following a minimal dose approach, based on multi-joint exercises [20, 21]. Before each session, 10 repetitions were performed at 30% of the 6RM load in each exercise as warm up. The participants were oriented to training to momentary concentric failure, as previously defined to control effort between conditions [22]. During training, the loads were adjusted between each set to allow momentary concentric failure to occur in the required repetition range (6 or 15RM). The participants were advised to perform the exercises with a controlled repetition duration, taking 2–3 s for each phase of movement and no pauses between muscle actions. The rest intervals between sets and exercises lasted for 2 min. Immediately after the training sessions, HRV, SBP, DBP and double product (DP) were collected. Two other measures were performed: 1 and 24 h after the session, always following the methodology adopted in the initial resting data collection. During the control session the participants followed the same procedures, but substituted the RT session for 20 min of rest in the laboratory. The time was stipulated according to the average duration of the RT sessions.

### Heart rate variability analysis

After completion of three test sessions, data obtained from each participant was transferred from the heart rate monitor through a transmission cable supplied by the device. Data processing and analysis were performed using Kubios HRV 3.0.2 software (© Kubios Oy, Finland). Artifacts such as peaks or discrepant intervals were manually extracted to correct possible errors in the values ​​analyzed. The parts of greater stability of the signal were selected for the analyzes, which included at least 256 consecutive beats [4]. The analyzes were made from linear time domain models: rMSSD (the square root of the mean squared differences of successive R–R intervals), and in the frequency domain, through the spectral analysis: low frequency components (LF) being representative of the sympathetic component of the system, high frequency (HF) being representative of the parasympathetic component of the system and the ratio LF/HF representing the sympatho-vagal balance. The HRV was also analyzed by nonlinear models from the Approximate Entropy (ApEn) analysis, which enabled the quantification of the sympathetic and parasympathetic components of the autonomic modulation of the heart rate.

### Data analysis

Data are presented as mean ± standard deviation. The values for the HRV, SBP, DBP and DP before and after the three experimental sessions were compared by repeated measures ANOVA with a 3 × 4 (protocol × time). If necessary, multiple comparisons with confidence adjustment by the Bonferroni procedure were used as post hoc analyses. The analyses were performed with IBM SPSS Statistics 21 software (SPSS Inc., Chicago, Illinois, USA). The main factors were the protocol (control, 6RM and 15RM) and the time (rest, after the session, 1 h after and 24 h after). The alpha value of p ≤ 0.05 was considered significant.

## Results

All participants completed the three protocols. The characteristics of the participants are presented in Table 1. According to the results of the randomization by lot, three women performed the control session first, nine of the 6RM session, and three the15RM session. The volume of work performed (sets × repetitions × load) in the different protocols are present in Table 2.

**Table 1:** Characteristics of subjects

Variables	Mean ± SD
Age (years)	57.73 ± 6.11
Body mass (kg)	65.77 ± 10.37
Heigth (m)	1.56 ± 0.08
Body mass index (kg/m^2^)	26.90 ± 3.74

*SD* standard deviation

**Table 2:** Work performed in different exercise protocols

Groups	Bench press	Pull down	Leg press	Total session work
6RM	305.33 ± 39.53	534.36 ± 98.29	2008 ± 406.38	2847.70 ± 508.67
15RM	535 ± 59.54	1037.66 ± 231.24	3743.53 ± 711.83	5316.20 ± 837.87

Values expressed as mean ± standard deviation. Work calculated as sets × repetition × load

### Blood pressure

The SBP and DBP data before and after the different RT protocols (control, 6RM and 15RM) are described in Table 3. Rest values were similar for all protocols (p ≥ 0.05). Compared with the resting values, there was an increase in SBP for the 6RM protocol immediately after RT (128 ± 17 vs. 140 ± 17 mmHg; p ≤ 0.05). There was no significant difference in DBP values immediately after RT between groups (78 ± 10 vs. 78 ± 11 mmHg, p ≥ 0.05). There were no differences in blood pressure between protocols at any other time point.

**Table 3:** Cardiovascular parameters at rest, immediately after, 1 h after and 24 h after the resistance training protocols

Variables	Groups	Rest	After	1 h	24 h
SBP (mmHg)	Control	132.26 ± 17.92	131.26 ± 17.48	133.73 ± 18.39	127.20 ± 14.30
	6RM	128.33 ± 17.07	140.33 ± 16.99*	130.86 ± 17.63	129.93 ± 16.07
	15RM	130.80 ± 21.22	137.06 ± 14.94	130.00 ± 17.55	128.26 ± 14.41
DBP (mmHg)	Control	78.06 ± 7.30	78.26 ± 8.31	79.73 ± 7.76	75.00 ± 8.76
	6RM	79.13 ± 9.58	77.60 ± 11.30	77.86 ± 10.82	76.20 ± 8.94
	15RM	77.00 ± 7.21	76.20 ± 11.02	77.13 ± 9.21	77.73 ± 9.35
HR (bpm)	Control	67.86 ± 6.63	65.98 ± 7.26	64.04 ± 8.26	70.67 ± 8.36
	6RM	66.00 ± 6.35†	76.24 ± 9.35†	68.59 ± 8.66	70.49 ± 7.71
	15RM	68.70 ± 9.17	85.32 ± 13.21†^‡^	73.82 ± 11.23†^‡^	72.06 ± 9.54
DP (mmHg bpm)	Control	8987.45 ± 1640.27	8650.36 ± 1477.97*	8544.24 ± 1544.29*	8981.66 ± 1391.14
	6RM	8467.05 ± 1422.90^†^	10,718.28 ± 2090.03*^†^	8972.57 ± 1756.20*	9128.50 ± 1280.63*
	15RM	9012.49 ± 2025.25	11,769.10 ± 2783.33*^†‡^	9608.14 ± 2212.70^†‡^	9211.75 ± 1377.20

Values expressed as mean ± standard deviation

*SBP* systolic blood pressure, *DBP* diastolic blood pressure, *HR* heart rate, *DP* double product

* Significantly different from pre-intervention (p ≤ 0.05). ^†^ Significantly different from the control session (p ≤ 0.05). ^‡^ Significantly different from the 6RM session (p ≤ 0.05)

### Heart rate

The HR data before and after the control, 6RM and 15RM protocols are described in Table 3. The results show a significantly higher HR after 6RM in comparison to control immediately after the exercise session: (76.24 ± 9.35 bpm vs 65.98 ± 7.26 bpm; p < 0.05). Heart rate was significantly higher, immediately (85.32 ± 13.21 bpm), and 1 h after (73.82 ± 11.23 bpm) in the 15RM protocol when compared to control (65.98 ± 7.26 and 6404 ± 8.26 bpm); and 6RM (76.24 ± 9.35 and 68.59 ± 8.66).

### Double product

The DP data before and after the different protocols (control, 6RM and 15RM) are described in Table 3. The results of the DP show significant differences in both 6RM: (10,718.28 ± 2090.03) and 15RM (11,769.10 ± 2783.33) immediately after the session in comparison to the control (8650.36 ± 1477.97); p < 0.05. The values for 15RM were significantly higher than both 6RM and control immediately after the session and 1 h after the session (p < 0.05). Within groups comparison revealed that there was a significant difference for groups 6RM and 15RM between baseline and immediately after exercise (p < 0.05).

### Heart rate variability

**Fig. 1:**
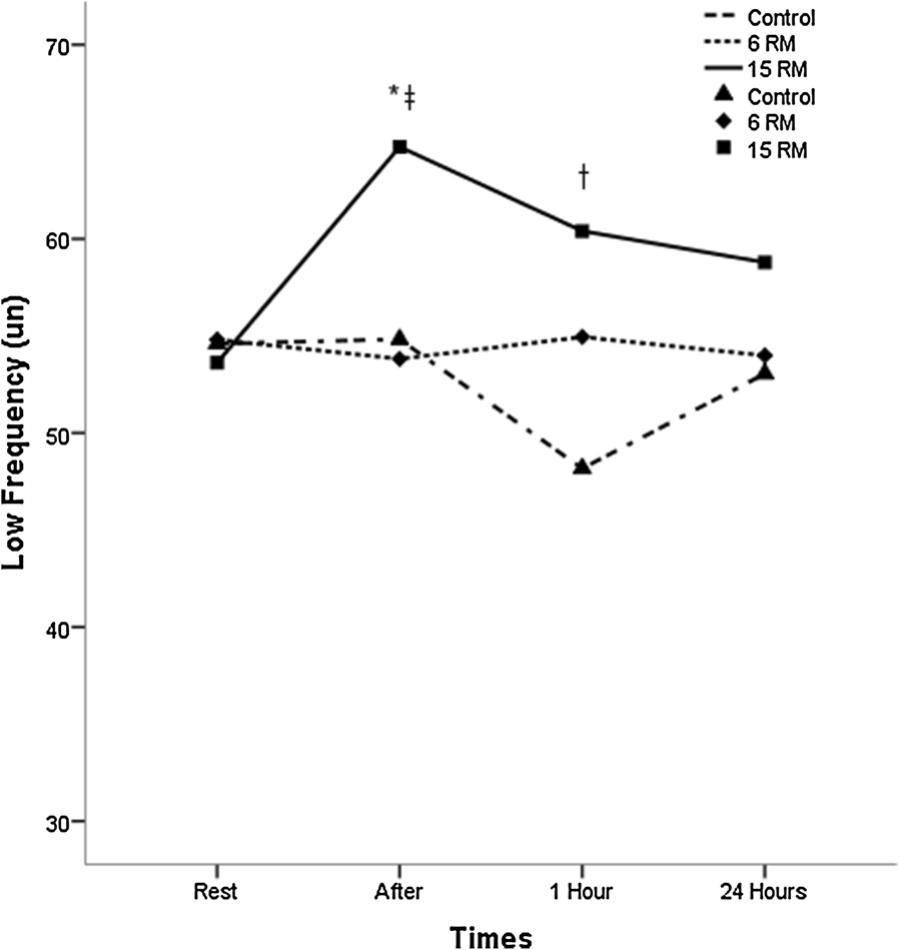
Changes observed in the low frequency values after the control sessions (triangles), 6RM (lozenges), 15RM (squares), where the time 1 rest, time 2 after the intervention, time 3 one hour after the intervention and time 4 twenty hours after the intervention. *Significantly different from pre-intervention (p ≤ 0.05). ^†^Significantly different from the control session (p ≤ 0.05). ^‡^ Significantly different from the 6RM session (p ≤ 0.05)

**Fig. 2:**
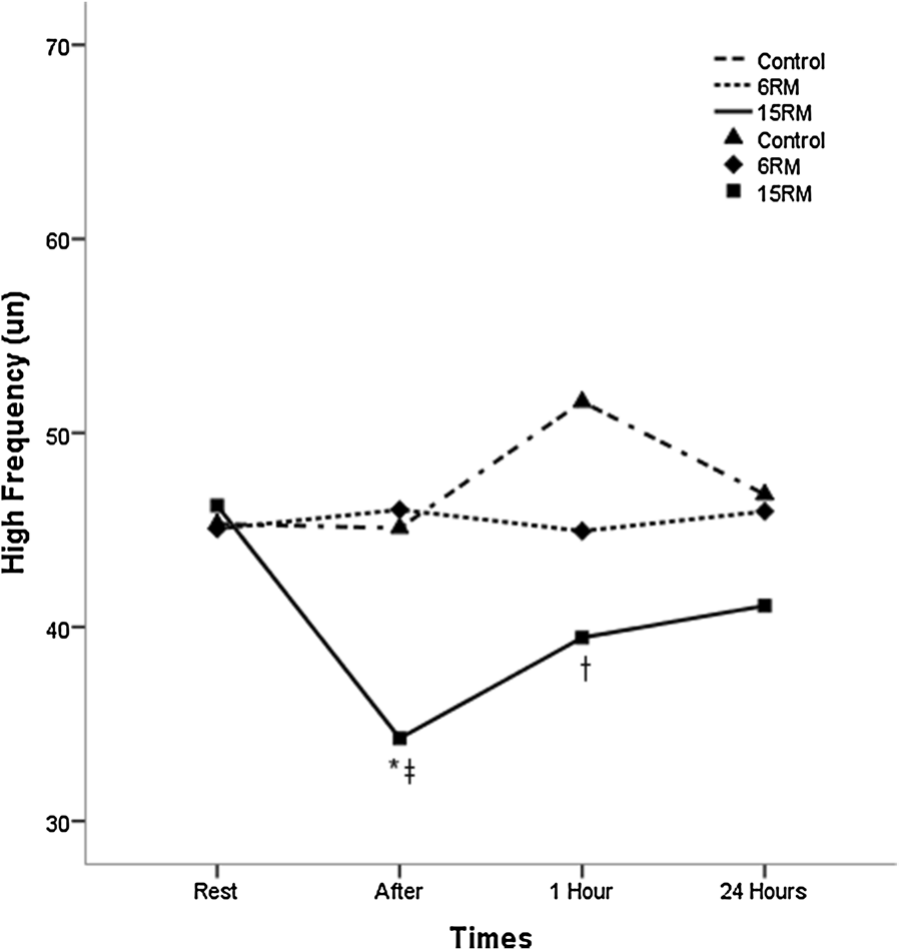
Changes observed in the high frequency values after the control sessions (triangles), 6RM (lozenges), 15RM (squares), where the time 1 rest, time 2 after the intervention, time 3 one hour after the intervention and time 4 twenty hours after the intervention. *Significantly different from pre-intervention (p ≤ 0.05). ^†^Significantly different from the control session (p ≤ 0.05). ^‡^ Significantly different from the 6RM session (p ≤ 0.05)

**Fig. 3:**
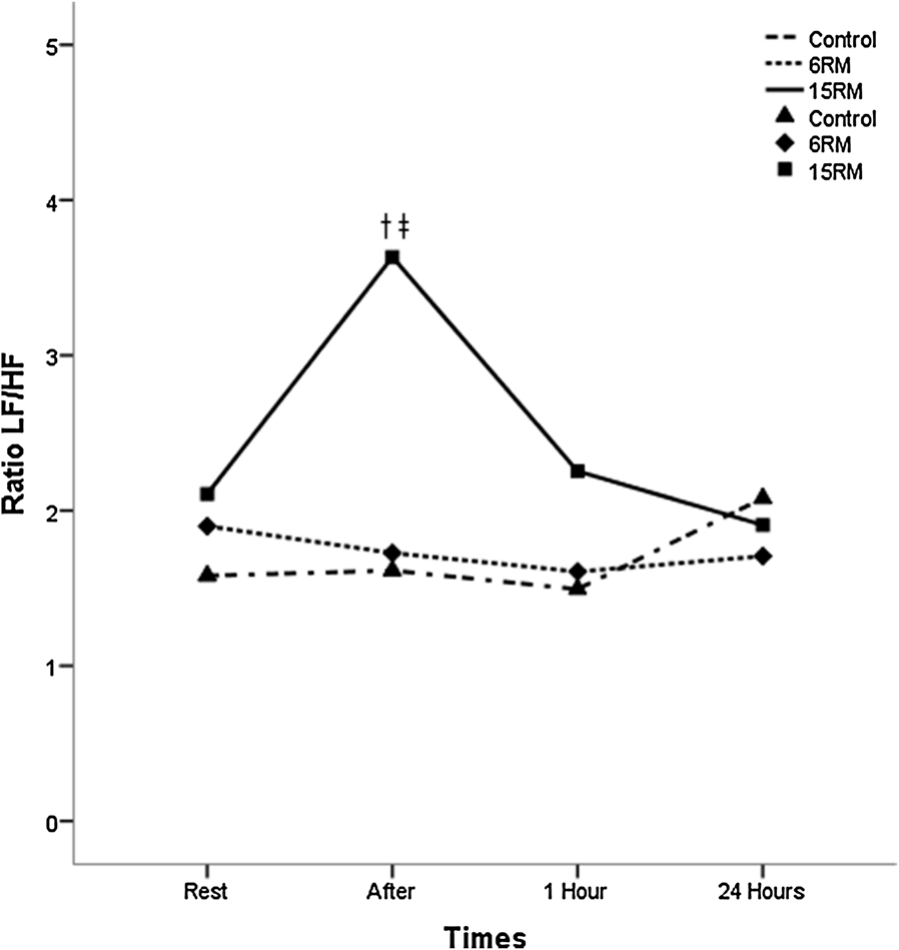
Changes observed in the ratio low frequency/high frequency values after the control sessions (triangles), 6RM (lozenges), 15RM (squares), where the time 1 rest, time 2 after the intervention, time 3 one hour after the intervention and time 4 twenty hours after the intervention. ^†^Significantly different from the control session (p ≤ 0.05). ^‡^Significantly different from the 6RM session (p ≤ 0.05)

The HRV indices are described in Table 4 and in Figs. 1, 2 and 3. Rest values were similar for all protocols in all variables (p ≥ 0.05). The 15RM protocol resulted in significantly lower values of rMSSD index immediately after the exercise session when compared to the 6RM and control protocols (p < 0.05). For the LF index there were no significant differences between the protocols of 6RM and control, however the 15RM protocol resulted in significantly higher values compared to control 1 h after session and to 6RM immediately after session (p < 0.05). In the HF index there was a contrary response; 15RM protocol resulted in significantly lower values when compared to the group control and 6RM (p < 0.05). The measurements of the LF/HF ratio showed a significant increase in the values for the 15RM protocol in relation to the others immediately after the session (p < 0.05). Within groups comparison revealed that there was a significant difference in the LF and HF for 15RM protocol immediately after exercise when compared to baseline (p < 0.05).

**Table 4:** Linear and non-linear parameters of the heart rate variability at rest, immediately after, 1 h after and 24 h after the resistance training protocols

Variables	Groups	Rest	After	1 h	24 h
rMSSD (ms)	Control	21.51 ± 14.26	27.38 ± 18.93*	33.50 ± 22.17*	28.60 ± 29.24
	6RM	26.50 ± 16.92	23.12 ± 18.58	22.24 ± 14.88*^†^	21.51 ± 14.97*
	15RM	21.26 ± 13.05	12.62 ± 16.33*†^‡^	17.74 ± 11.16^†^	24.08 ± 22.91
LF (μn)	Control	54.60 ± 18.55	54.82 ± 17.82	48.17 ± 22.15	53.05 ± 26.47
	6RM	54.80 ± 22.56	53.82 ± 21.16	54.94 ± 20.11	53.99 ± 19.24
	15RM	53.62 ± 22.88	64.74 ± 20.68*^‡^	60.40 ± 18.07^†^	58.78 ± 18.16
HF (μn)	Control	45.30 ± 18.61	45.10 ± 17.83	51.60 ± 21.95	46.83 ± 26.40
	6RM	45.06 ± 22.62	46.04 ± 21.11	44.94 ± 20.07	45.96 ± 19.21
	15RM	46.26 ± 22.82	34.26 ± 21.39*^‡^	39.45 ± 18.04^†^	41.09 ± 18.10
LF/HF	Control	1.58 ± 1.20	1.61 ± 1.35	1.49 ± 1.75	2.08 ± 2.27
	6RM	1.90 ± 1.72	1.72 ± 1.49	1.60 ± 1.11	1.70 ± 1.72
	15RM	2.10 ± 2.76	3.63 ± 3.32^†‡^	2.25 ± 2.06	1.90 ± 1.38
ApEn	Control	1.14 ± 0.13	1.14 ± 0.15	1.07 ± 0.16	1.11 ± 0.16
	6RM	1.14 ± 0.16	1.17 ± 0.23	1.09 ± 0.17	1.16 ± 0.19
	15RM	1.16 ± 0.10	1.22 ± 0.20	1.17 ± 0.08^†‡^	1.12 ± 0.18

Values expressed as mean ± standard deviation. rMSSD square root of the square mean of the differences between the adjacent normal RR intervals, expressed in milliseconds, high frequency HF, expressed in standard units, LF low frequency, expressed in normalized units, LF/HF ratio high/low frequency, Ap En approximate Entropy

* Significantly different from pre-intervention (p ≤ 0.05). ^†^ Significantly different from the control session (p ≤ 0.05). ^‡^ Significantly different from the 6RM session (p ≤ 0.05)

## Discussion

The results of the present study suggest that the performance of a session of high intensity of effort RT using lighter loads and a higher number of repetitions (15RM) promoted greater changes in cardiovascular parameters such as DP and HR, indicating greater cardiovascular stress, when compared to 6RM and control. On the other hand, the performance of a high intensity of effort RT session using heavier loads and fewer repetitions (6RM) did not change the analyzed cardiovascular parameters when compared to a control condition. Therefore, despite the two protocols involving high intensity of effort, the heavier load and the fewer repetition protocol (6RM) induced lower cardiovascular stress and therefore might be considered a safer alternative, particularly for those with AH.

The use of HRV parameters as an indication of cardiovascular risk is already well established [4]. However, its relationship with the practice of RT in people with hypertension is not well understood. Some authors, such as Lima et al. [23] and Rezk et al. [24], suggest that RT with heavier loads leads to an increase in sympathetic activation due to the greater mechanical overload in the vascular system [25], with consequent decrease in HRV. However, our results showed that HRV components that indicate sympathetic activation were greater after RT performed at lighter loads and a higher repetition range (15RM). According to our results, immediately after the 15RM protocol, there was an increase in the sympathetic predominance, as demonstrated by the variables LF and LF/HF, and a decrease in the parasympathetic predominance as demonstrated by the rMSSD and HF variables. Regarding HR and DP, the values for 15RM were greater than the control and 6RM protocols. On the other hand, the 6RM protocol did not result in significant changes in HRV when compared to the control condition. This suggests that load might not influence sympathovagal balance. Other variables such as total work volume and time under tension might be related to the sympathetic activation system and consequent increase in cardiovascular risk, as previously suggested [23, 24].

Despite the recent evidence presenting RT as an alternative for the treatment of several comorbidities, including cardiovascular diseases [26, 27], its prescription is often neglected for patients with AH in many guidelines [28, 29]. When RT is recommended, the guidance is to perform it using lighter loads and a higher number of repetitions, arguing that heavier load RT would not be safe [30–32]. Our results challenge this suggestion, since after RT using heavier loads, the responses of variables such as BP and HRV were similar to those at rest. On the other hand, the practice of RT with lighter loads resulted in an increase in DP response and a decrease in HRV immediately after its execution, with these changes persisting even 1 h after the session end. Chronic studies are warranted to analyze the long-term effect of different RT protocol on cardiovascular function in order to test if this acute effects translate into chronic results.

RT might have several benefits for patients with hypertension, such as decreasing resting BP [26, 27, 33] and increasing muscle strength. As for the last, it has been previously reported that higher levels of muscle strength are associated with lower mortality rates both in the general population [34–36], and in people with AH [37]. Therefore, increasing muscle strength might be an important aim of RT protocols. Considering that previous studies showed that training with a higher or lower number of repetitions results in similar strength gains, when performed to failure [38–41], and further, even microvascular adaptations appear to be similar whether using heavier or lighter loads [42] the choice of protocol might be based on other aspects, such as safety and discomfort. In this regard, previous studies reported that the practice of RT with lighter loads and greater number of repetitions generates greater discomfort when compared to RT with heavier loads and fewer repetitions [39, 43]. Moreover, previous studies have shown that performing RT with heavier loads and a lower number of repetitions resulted in smaller increases in blood pressure and pulse rate during training when compared with training at lighter loads and higher number of repetitions [44–46]. When combined with these previous findings, our results suggest that the protocols with a heavier load and lower repetitions might be recommended to promote increases in muscle strength and improve health parameters in hypertensive patients while resulting in a reduced cardiovascular overload.

## Conclusion

In conclusion, performing RT with lower loads and a higher number of repetitions seems to promote acute increases in sympathetic ANS activity, which may be related to cardiovascular stress. On the other hand, heavier load and lower repetition RT did not significantly impact upon autonomic modulation when compared to a control session. The study is not without limitations, such as, the absence of BP and HRV measurement during the protocols. However, there are reasonable amounts of evidence comparing different protocols on BP during exercise, as previously cited. As for HRV, it is not possible to reliably measure it during exercise by currently available methods; however, it is reasonable to suggest that, if it is altered after the interruption of the exercises, similar dynamics might be seen during exercise. Future studies should confirm if the present findings are reproduced in different populations and also evaluate the long term effects of different protocols in order to allow a better insight into the risk–benefit ratio of such approaches with respect to health outcomes and adverse events.

## Abbreviations

AH: arterial hypertension
BP: blood pressure
ANS: autonomic nervous system
HRV: heart rate variability
R–R intervals: intervals between beats
RT: resistance training
RM: repetitions maximum
BMI: body mass index
SPB: systolic blood pressure
DPB: diastolic blood pressure
HR: heart rate
DP: double product
rMSSD: square root of the mean squared differences of successive R–R intervals
LF: low frequency
HF: high frequency
LF/HF: ratio low frequency high frequency
ApEn: approximate entropy
